# Identification of the likely translational start of *Mycobacterium tuberculosis* GyrB

**DOI:** 10.1186/1756-0500-6-274

**Published:** 2013-07-15

**Authors:** Shantanu Karkare, Amanda C Brown, Tanya Parish, Anthony Maxwell

**Affiliations:** 1 Department of Biological Chemistry, John Innes Centre Norwich Research Park, Norwich NR4 7UH, UK; 2 Barts and The London School of Medicine and Dentistry, Queen Mary University of London, 4 Newark Street, London E1 2AT, UK; 3 Present address: Novartis Institute of Biomedical Research, Basel 4056, Switzerland; 4 Present address: Oxford Gene Technology, Begbroke Science Park, Begbroke Hill, Woodstock Road, Begbroke OX5 1PF, UK

**Keywords:** Gyrase, Topoisomerase, *Mycobacterium tuberculosis*

## Abstract

**Background:**

Bacterial DNA gyrase is a validated target for antibacterial chemotherapy. It consists of two subunits, GyrA and GyrB, which form an A_2_B_2_ complex in the active enzyme. Sequence alignment of *Mycobacterium tuberculosis* GyrB with other bacterial GyrBs predicts the presence of 40 potential additional amino acids at the GyrB N-terminus. There are discrepancies between the *M. tuberculosis* GyrB sequences retrieved from different databases, including sequences annotated with or without the additional 40 amino acids. This has resulted in differences in the GyrB sequence numbering that has led to the reporting of previously known fluoroquinolone-resistance mutations as novel mutations.

**Findings:**

We have expressed *M. tuberculosis* GyrB with and without the extra 40 amino acids in *Escherichia coli* and shown that both can be produced as soluble, active proteins. Supercoiling and other assays of the two proteins show no differences, suggesting that the additional 40 amino acids have no effect on the enzyme *in vitro*. RT-PCR analysis of *M. tuberculosis* mRNA shows that transcripts that could yield both the longer and shorter protein are present. However, promoter analysis showed that only the promoter elements leading to the shorter GyrB (lacking the additional 40 amino acids) had significant activity.

**Conclusion:**

We conclude that the most probable translational start codon for *M. tuberculosis* GyrB is GTG (Val) which results in translation of a protein of 674 amino acids (74 kDa).

## Findings

### Introduction

Bacterial DNA gyrase is a validated target for antibacterial chemotherapy [1]. It is a member of the DNA topoisomerase family of enzymes, which are responsible for maintaining and manipulating the topological state of DNA [2,3]. These enzymes are required for vital processes such as DNA replication, transcription, recombination and chromatin remodelling. Topoisomerases can be classified into two types, I and II, dependent on whether their reactions involve transient cleavage of one (I) or both (II) strands of DNA. Due to the important role played by topoisomerases in maintaining cell viability, they are attractive clinical targets for chemotherapeutics [1,4,5].

DNA gyrase is a type II topoisomerase that consists of two subunits, GyrA and GyrB, which form an A_2_B_2_ complex in the active enzyme [1,6]. Gyrase introduces negative supercoils into DNA, in addition to catalysing relaxation and decatenation. The supercoiling and decatenation reactions require ATP hydrolysis, which occurs in the GyrB subunits. The absence of gyrase in most eukaryotes and its essentiality in bacteria have made it an ideal target for antimicrobial agents [1].

Tuberculosis (TB) is the world’s most deadly bacterial disease with around a third of the world’s population infected and over 1 million deaths every year [7]. Although treatments are available, drug-resistant strains, MDR (multi-drug resistant) and XDR (extensively drug resistant) TB, pose serious problems. Moxifloxacin, a fluoroquinolone that targets DNA gyrase, has been successfully used against *Mycobacterium tuberculosis*, particularly MDR strains [8]. The search for new anti-TB agents involves the further exploitation of *M. tuberculosis* gyrase as a target for TB therapy and a need to achieve a greater understanding of this enzyme.

Most of our current information about gyrase concerns the enzyme from *Escherichia coli*, a Gram-negative bacterium, with limited information about gyrase from *M. tuberculosis*[9], which is generally described as a Gram-positive bacterium. In *M. tuberculosis* the two genes encoding DNA gyrase, *gyrB* and *gyrA*, are located adjacent to each other, and although there is a separate promoter for *gyrA* (P_A_), the primary transcript appears to be dicistronic [10]. Analysis of the region upstream of *gyrB* suggests there are multiple promoters, but it appears that most of the transcripts originate from P_B1_ with the others potentially involved in the fine-tuning of transcription [10].

There are significant discrepancies in the *M. tuberculosis* GyrB sequences retrieved from different databases. For example, in the case of NCBI code CAB02426 for *M. tuberculosis* H37Rv GyrB, 40 additional amino acids are present in the sequence starting with amino acids MGKNEARRSA. While the same protein cross-referenced in UniProtKB/Swiss-Prot as P0C5C5 lacks these additional amino acids; it is 674 amino acids and starts with the amino acid sequence: MAAQKKKAQD. This is not uniform across UniProtKB/Swiss-Prot as in other *M. tuberculosis* strains, such as *M. tuberculosis* T92, the GyrB sequence (UniProtKB/Swiss-Prot: D5XPA3) also has the 40 additional amino acids and starts with MGKNEARRSA. Similarly, in the case of *M. bovis* AF2122/97 both the NCBI (CAD92867) and UniProtKB/Swiss-Prot (Q7U312) databases indicate the presence of an additional 40 amino acids at the GyrB N-terminus. Re-annotation of the *M. tuberculosis* H37Rv gene sequence [11] suggested that GyrB is a protein of 714 amino acids and MW 78.4 kDa, These various discrepancies in GyrB sequence annotation have led to differences in sequence numbering, which in turn has resulted in the reporting of previously known fluoroquinolone resistance mutations as novel mutations [12].

In this paper we investigate the start codon for *M. tuberculosis* GyrB sequence by promoter and transcript analysis, and studies on the expressed protein.

### Materials and methods

#### ***Preparation and purification of proteins***

*M. tuberculosis gyrB* sequences were amplified from *M. tuberculosis* genomic DNA and cloned into TOPO® cloning vectors followed by sub-cloning into pET20 using the Clonable^TM^ Ligation kit (Novagen). Expression and purification of the GyrB and GyrA proteins were as previously described [13].

#### ***Enzyme assays***

*M. tuberculosis* DNA gyrase assays (supercoiling, relaxation and decatenation) were performed as described previously [13]. Peptide mass fingerprinting and mass spectrometry analyses were carried out by Gerhard Saalbach (John Innes Centre Proteomics Shared Facility).

#### ***RT-PCR***

**Figure 1:**
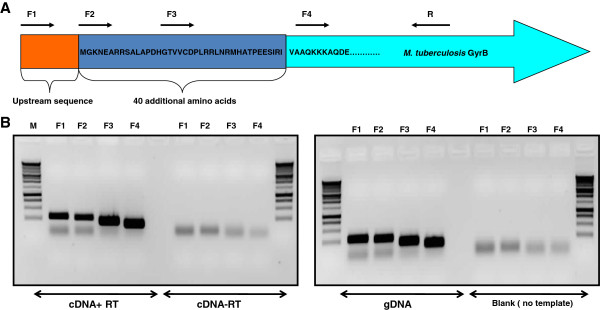
**Identification of mRNA transcripts for *M. tuberculosis gyrB*. (A)** Schematic showing the position of primers (F1, F2, F3, F4, and R) used for RT-PCR. **(B)** Products from RT-PCR reactions were analysed by agarose gel electrophoresis. Left primers used are indicated above the lanes and correspond to Figure 1A; M – markers (1 kb ladder); cDNA+RT: plus reverse transcriptase; cDNA-RT: no reverse transcriptase; gDNA: genomic DNA; blank: no template control.

Total RNA was extracted from standing cultures of *M. tuberculosis* H37Rv, as previously described [14], and DNase treated prior to cDNA synthesis [15]. cDNA was synthesized from 1 μg of total RNA using SuperScript II RT (Invitrogen) and random primers (Invitrogen) according to the manufacturer’s instructions; an RT-minus control for each sample was also included and processed in tandem. To identify which transcripts were present, four forward primers were designed: F1 (5′-CAC GGC GCG GTT AGA TGG GTA A-3’), which binds near the start of the region corresponding to the additional 40 amino acids at nucleotide positions 5109–5130 [16]; F2 (5′-TGG GTA AAA ACG AGG CCA GAA GAT C-3′) corresponds to the start of the region encoding the additional 40 amino acids at positions 5124–5148; F3 (5′-CGA CTC AAC CGC ATG CAC GCA-3′), which corresponds to the middle of the region comprising the 40 amino acids at position 5195–5215; F4 (5′-CCA GAA AAA GAA GGC CCA AG-3′), which corresponds to 5 nucleotides after the end of the 40 amino acids at positions 5248–5267; these were used in combination with a single reverse primer R (5′-ATA ACC GGC CAT CGC CTC GT-3′), as shown in Figure 1A. RT-PCR reaction mixes contained GoTaq PCR Mastermix (Promega), 10 pmol forward primer, 10 pmol reverse primer R, and 100 ng cDNA. Cycling conditions were: 25 cycles of 94°C for 30“, 56°C for 30”, and 72°C for 1’, followed by 72°C for 5’ final extension. The amplified products were run on a 1% agarose gel and visualized with ethidium bromide staining.

#### ***Promoter analysis***

Primer pairs Met-F: 5′-AGT ACT CAC GTC GAT CGG CCC AGA ACA AGG CGC- 3′ and Met-R: 5′-CCC GGG CAT CTA ACC GCG CCG TGC-3′ or Val-F: 5′-AGT ACT CAC GTC GAT CGG CCC AGA ACA AGG CGC-3′ and Val-R: 5′-CCC GGG CAC GAT CCG AAT ACT CTC CTC AGG G-3′ were used to amplify the upstream regions. The thermocycler program used was: 2 min denaturation at 94°C, 25 cycles of 94°C for 15’, 55°C for 1’, and 72°C for 2’, with a final extension at 72°C for 10’. PCR products were cloned into the pGEM-T easy vector (Promega), sequence verified, and sub-cloned as *Sca*I fragments into pSM128 [17] to construct the plasmids, which were called: pSM128-Met, carrying 128 bp upstream of the predicted transcriptional start site (nucleotides 4998–5125, ending with Met); and pSM128-Val (4998–5242) carrying a larger 245 bp upstream region ending with Val (GTG). Plasmids were introduced into *M. tuberculosis H37Rv* by electroporation and selected on streptomycin. Three individual transformants were inoculated into 10 ml of standing cultures (7H9-Tw medium Middlebrook 7H9 liquid medium supplemented with 10% (vol/vol) OADC (oleic acid, bovine serum albumin, d-glucose, catalase; Becton Dickinson) and 0.05% (wt/vol) Tween 80). Cell-free extracts were prepared and β-galactosidase assays were performed, as previously described [18].

### Results

#### ***Sequence alignment of M. tuberculosis GyrB***

**Figure 2:**
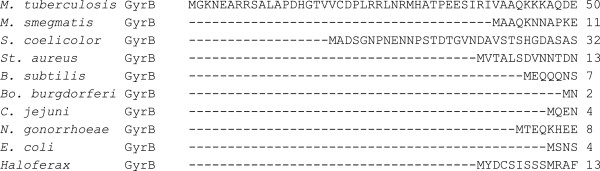
**Multiple sequence alignment of bacterial GyrBs.** Amino acid sequence alignment of *Mycobacterium tuberculosis* GyrB (NCBI sequence) with other bacterial GyrBs using ClustalW 1.83 [19]. *S. coelicolor* = *Streptomyces coelicolor*; *St. aureus* = *Staphylococcus aureus*; *B. subtilis* = *Bacillus subtilis*; *Bo. burgdorferi* = *Borrelia burgdorferi*; *C. jejuni* = *Campylobacter jejuni*; *N. gonorrhoeae* = *Neisseria gonorrhoeae*; *E. coli* = *Escherichia coli*.

As established previously [12] there have been several numbering systems reported for *M. tuberculosis* GyrB, which has led to considerable confusion in relation to the location of quinolone-resistance mutations. Figure 2 shows a sequence alignment of *M. tuberculosis* GyrB with other bacterial GyrBs, highlighting a stretch of 40 amino acids at the N-terminus, the presence of which has contributed to the confusion in amino acid numbering. We set out to establish the significance of these additional amino acids and whether the protein produced in *M. tuberculosis* includes this sequence.

#### ***Enzyme activity of GyrBs with and without the additional 40 amino acids***

Expression plasmids (based on pET20) were constructed for production of GyrBs with and without the additional 40 amino acids, and the resulting proteins were purified by affinity chromatography and by gel filtration. The presence of the additional 40 amino acids in the longer GyrB was verified by peptide mass fingerprinting and mass spectrometry analysis (data not shown).

The two GyrB proteins were complexed with *M. tuberculosis* GyrA and the supercoiling, relaxation and decatenation activities were measured as previously described [13]. We found that there was no significant effect of the additional amino acids on these activities, for example the supercoiling activities of the two enzymes are nearly identical (Additional file 1: Figure S1), and no effect on the sensitivity of the enzyme to novobiocin (data not shown); the binding site for novobiocin lies in the N-terminal domain of GyrB [1]. This strongly suggests, *in vitro* at least, that the additional 40 amino acids are of no functional significance.

#### ***Identification of the M. tuberculosis gyrB transcript***

We wanted to determine whether GyrB was produced as the longer or shorter protein; as a first step we analysed the transcripts. RT-PCR was performed to determine which GyrB mRNA transcripts are produced in *M. tuberculosis* under normal aerobic growth. Primers were designed to distinguish between longer and shorter transcripts which could encode for longer or shorter proteins respectively (Figure 1A). As shown in Figure 1B, PCR products were obtained with all primer sets (F1, F2, F3, F4) indicating that mRNA was being produced that could potentially encode the longer protein. The identity of the products obtained was confirmed by sequencing. This RT-PCR experiment indicates that the nucleotide sequence that could encode the 40 amino acids is transcribed.

#### ***Promoter analysis***

**Figure 3:**
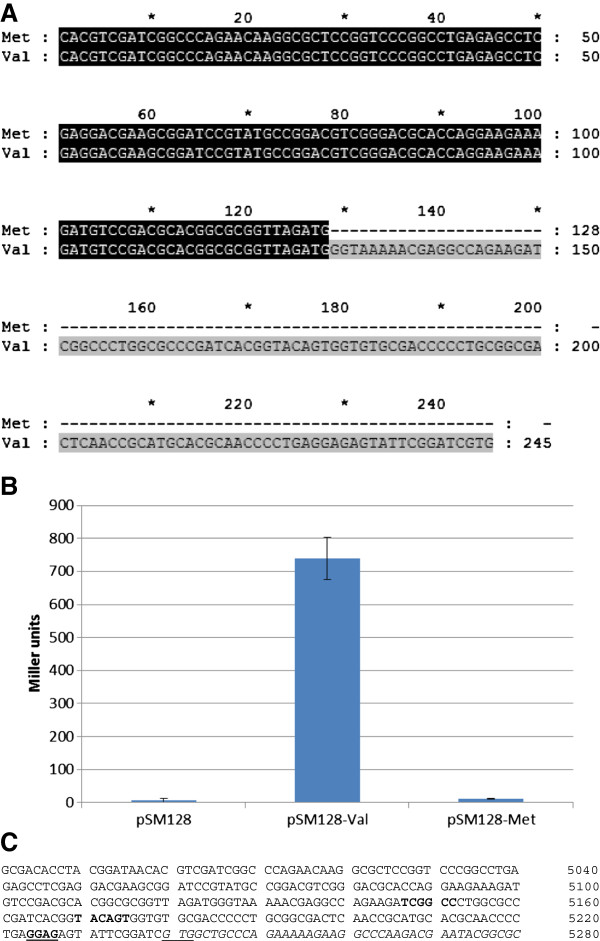
**Promoter activity determined in *M. tuberculosis*. (A)** Upstream regions of GyrB ending in Met and Val were cloned into pSM128 to give pSM128-Met and pSM128-Val respectively. **(B)***M. tuberculosis* recombinants were grown as described in Materials and Methods, and β-galactosidase activity measured. Transformants were obtained for pSM128 (empty vector control); pSM128-Val (containing *M. tuberculosis* nucleotides 4998–5125, upstream of the ORF for GyrB) and pSM128-Met (containing nucleotides 4998–5242). Data are mean +/- standard deviation from three independent transformants tested in duplicate. **(C)***M. tuberculosis* genome sequence 4981–5280 that includes the start of *gyrB* and its upstream sequence. The predicted ribosome binding site is in bold and underlined; the predicted promoter elements are in bold; the *gyrB* coding sequence is in italics with the Val start codon underlined. The annotations are consistent with earlier work [10].

From the enzyme activity assays above it appears that the additional 40 amino acids at the N-terminus of GyrB have no functional significance, but the mRNA analysis suggests the possibility that there are RNA species that can encode these 40 amino acids. In order to identify the translational start site, we analysed expression of the LacZ reporter gene in *M. tuberculosis.* Two plasmids were tested for activity: pSM128-Met contained the region upstream of the additional 40 amino acids terminating at ATG (5125), and pSM128-Val carrying the region upstream of the shorter GyrB terminating at GTG (5242) (Figure 3). Plasmid pSM128-Met showed no significant promoter activity (Figure 3B), while pSM128-Val demonstrated high level promoter activity (740 MU ± 63). (A comparable series of experiments involving the introduction of these plasmids into *M. smegmatis* gave very similar results; data not shown). These data suggest that, under these growth conditions, the expression of GyrB is from a promoter located upstream of the valine start codon and that the shorter protein is produced, i.e. without the additional 40 amino acids.

### Discussion

Sequence alignments of GyrBs from different bacterial species suggest that 40 additional amino acids could potentially be present at the *M. tuberculosis* GyrB N-terminus (Figure 2). In order to study the effect of these 40 amino acids on enzyme activity, we expressed and purified GyrB proteins in which the 40 amino acids were present or absent and found that there is no apparent effect of the additional amino acids on gyrase activity, as judged by supercoiling, relaxation and decatenation assays *in vitro*.

RT-PCR experiments were performed, which showed that the RNA sequence corresponding to the additional 40 amino acids at the GyrB N-terminus is present at the transcript level (Figure 1). However, promoter assays (Figure 3B) showed that only the shorter GyrB would be expressed at a significant level *in vivo*. These experiments suggest that the promoter elements are present in the region corresponding to the predicted 40 amino acids at the GyrB N-terminus (Figure 3C). The presence of transcripts containing the 40 amino acid region indicates possible post-transcriptional events occurring before the mature GyrB is formed.

### Conclusion

Taken together our results support *M. tuberculosis* GyrB being a protein of 74,058.7 Da (674 amino acids) that begins with the codon GTG (Val) at position 5240–5242 in the *M. tuberculosis* genome sequence [16]. A recent paper also supports this conclusion [12].

## Competing interests

The authors declare that they have no competing interests.

## Authors’ contributions

SK, ACB, TP and AM conceived the research ideas for the work. SK and ACB carried out the experiments. TP and AM supervised the work. SK, ACB, TP and AM wrote the manuscript. All authors read and approved the manuscript.

## Supplementary Material

Additional file 1: Figure S1 Supercoiling assays of short and long GyrBs. DNA supercoiling assays were carried out as described previously [13] with *M. tuberculosis* GyrB proteins, with (+40) and without (-40) the additional 40 amino acids, complexed with GyrA. Enzyme concentrations are indicated above the gel tracks and the topological forms of plasmid pBR322 are indicated at the side: OC = nicked circular; Rel = relaxed; SC = supercoiled. Click here for file
