# Supplementary material for: Identification of the likely translational start of Mycobacterium tuberculosis GyrB
Source: BMC Res Notes. 2013 Jul 15;6:274. doi: 10.1186/1756-0500-6-274 (PMC3724585; doi:10.1186/1756-0500-6-274)

**Additional Figure Legend**

**Additional file 1: Figure S1. Supercoiling assays of short and long GyrBs.** DNA supercoiling assays were carried out as described previously with *M. tuberculosis* GyrB proteins, with (+40) and without (-40) the additional 40 amino acids, complexed with GyrA. Enzyme concentrations are indicated above the gel tracks and the topological forms of plasmid pBR322 are indicated at the side: OC = nicked circular; Rel = relaxed; SC = supercoiled.


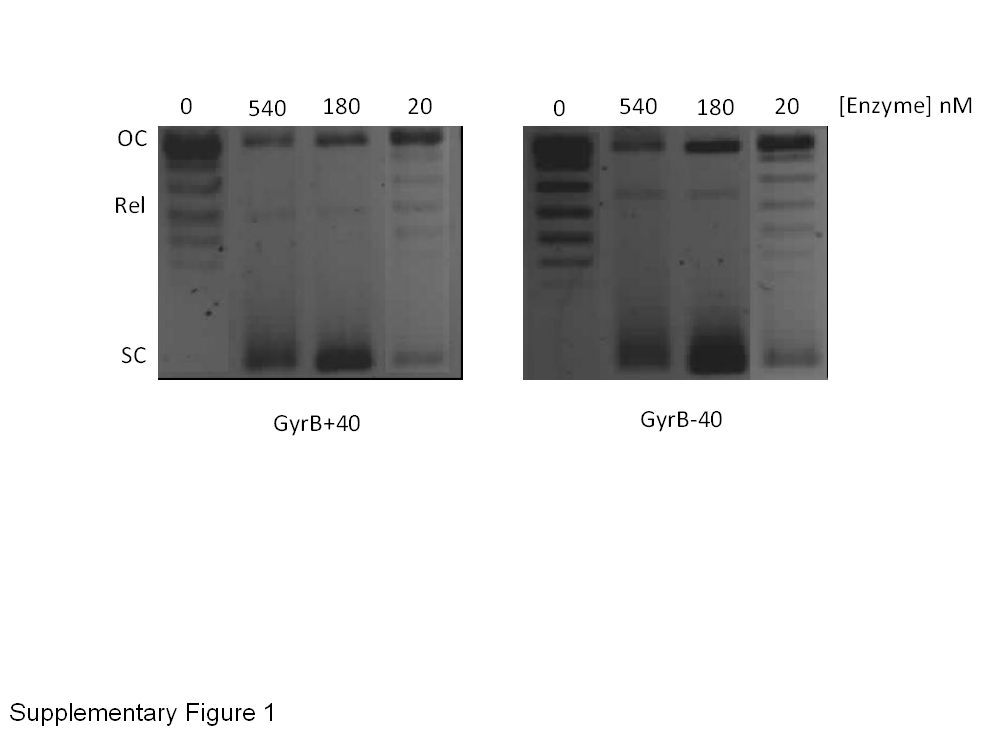

Supplement: Additional file 1: Figure S1 — Supercoiling assays of short and long GyrBs. DNA supercoiling assays were carried out as described previously [13] with M. tuberculosis GyrB proteins, with (+40) and without (-40) the additional 40 amino acids, complexed with GyrA. Enzyme concentrations are indicated above the gel tracks and the topological forms of plasmid pBR322 are indicated at the side: OC = nicked circular; Rel = relaxed; SC = supercoiled. [file 1756-0500-6-274-S1.doc]
